# I_2_-SDS-H_2_O System: A highly Efficient Dual Catalytic Green System for Deprotection of Imines and *in Situ* Preparation of Bis(indolyl)alkanes from Indoles in Water

**DOI:** 10.5402/2012/635835

**Published:** 2012-08-27

**Authors:** Parasa Hazarika, Pallab Pahari, Manash Jyoti Borah, Dilip Konwar

**Affiliations:** ^1^Department of Chemistry, Jorhat Institute of Science and Technology, Assam Jorhat 785010, India; ^2^Synthetic Organic Chemistry Division, North East Institute of Science and Technology, Assam Jorhat 785006, India

## Abstract

A novel catalytic system consisting of I_2_-SDS-H_2_O has been developed which cleaves 2,3-diaza-1,3-butadiene, 1-aza-1,3-butadienes, oximes and in presence of indoles in the medium uses the corresponding aldehyde products to produce bis(indolyl)alkanes *in situ*. This one pot simple and mild dual catalytic system works in water at room temperature under neutral conditions.

## 1. Introduction

Using water as solvent in the organic reactions is one of the most important targets to organic chemists because of the easy availability, nontoxicity, and ecofriendly nature of the water [1–7]. In this endeavour, a number of chemical reactions such as Diels Alder, hetero Diels-Alder, 1,3-dipolar cycloaddition, oxidations, reductions, and others are performed successfully in water [1–3]. Also, it is reported that in few cases addition of the water increases the rate and the yield of a reaction and also enhances the enantioselectivity in a chiral synthesis [8]. But the main problems associated with water as a solvent is its poor ability to solubilise organic reactants and incapability to create anhydrous condition for moisture sensitive organic compounds and catalysts. To overcome the solubility problem, generally a surfactant is introduced to the reaction mixture. The surfactant, due to its hydrophobic and hydrophilic nature, forms micelles with water insoluble organic compounds and promote the desired reactions to occur inside the hydrophobic ambience of the micelle core [9, 10].

Cleavage of the C=N bonds is a very important transformation in organic synthesis as the C=N functionality is widely used to protect both the carbonyl and amines. There are a number of methods used for the cleavage of C=N bonds which include acidic reagents [11–13], oxidizing agents [14], metallic salts [15, 16], (PhSeO)_2_O [17], NaHSO_3_ [18], and others. Most of these methodologies suffer from serious drawbacks like involvement of strong Lewis and Bronsted acids, use of toxic and costly transition metals (i.e., Cr, Pd, Co), low temperature, longer reaction time, low yield, and difficulties in isolating the products. Therefore, development of efficient, mild and environment friendly reagents are always necessary. On the other hand, bisindoles are recently emerging as extremely important class of compounds because of their novel antibacterial and anticancer activities [19–21]. That is why a number of methodologies have also been postulated for the synthesis of bisindoles [22–29].

In our previous communications, we reported that surfactant- (SDS-) mediated cleavage of C=N bonds could be achieved with acetic anhydride [30] and surfactant-I_2_-water can be used for the deprotection of imines to carbonyls [31]. We have also shown that bis- and tris (indolyl)alkanes can be synthesized in presence of Bronsted acid in water [32]. In continuation of our research in hydrated media, herein, we wish to disclose the dual catalytic activity of the system I_2_-SDS-H_2_O which behaves as a Lewis acid [33] for the cleavage of 2,3-diaza-1,3-butadiene, 1-aza-1,3-butadienes, and oximes to produce carbonyls and amines, and the resulting reaction mixture reacts with indoles to produce bis (indolyl)alkanes *in situ* at room temperature under neutral conditions.

## 2. Results and Discussion

**Scheme 1:**
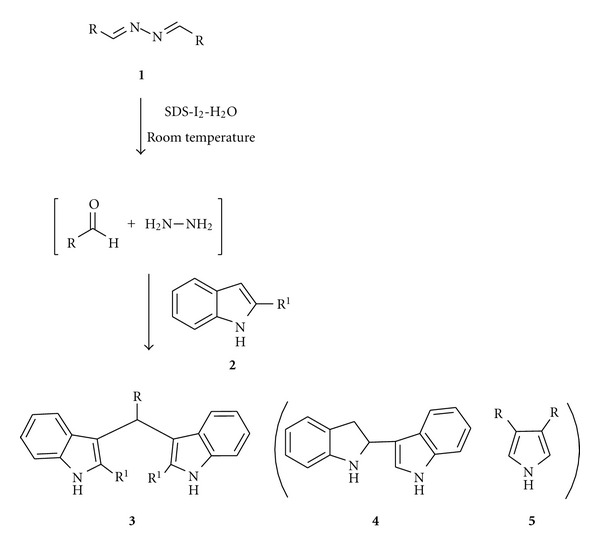
One pot synthesis of bisindoles from protected imine and indole.

For an initial study, molecular iodine was added to a mixture of 1,4-diphenyl-2,3-diaza-1,3-butadiene (**1a**) (1 mmol) and indole (**2a**) (2 mmol) in water. It was presumed that the activated imine should produce carbonyl compound in the reaction mixture which might be trapped with indole. But only a trace amount of 3,3′-bis(indolyl)phenylmethane (**3a**) was found to be formed in the reaction. We envisioned that the poor yield of the product may be due to the insolubility of organic substrates in water. Accordingly, we added a surfactant (SDS) to the reaction flask. To our delight, the reaction produced isolable amount of **3a** as a brown solid but half of the 1,4-diphenyl-2,3-diaza-1,3-butadiene (**1a**) left unreacted in the reaction mixture. So amount of indole (**2a**) was doubled (4 mmol) and the same reaction condition successfully produced quantitative amount of **3a**. The product was filtered out and the filtrate was treated with freshly distilled benzaldehyde (0.05 mmol) in ethanol which furnished **1a** (m.p. 91°C; lit [34] m.p. 92-93°C). This infers the simultaneous involvement of two C=N bonds of bis-anils, leaving behind hydrazine in the reaction mixture. Also, no self-reaction of individual starting materials leading to indole dimer [35] **(4)** or pyrrole formation [36] **(5)** were observed under the same reaction conditions (Scheme 1). Optimization of the reaction conditions was undertaken by employing different catalyst loadings under various surfactant conditions. The results are summarized in Table 1. It was found that the best result was obtained by the application of 15 mol% of I_2_ containing sodium dodecyl sulphate (SDS) and water at room temperature (entry 1, Table 1). In absence of the catalyst no formation of **3a** was observed even after stirring for 24 hours (entry 5, Table 1).

**Scheme 2:**
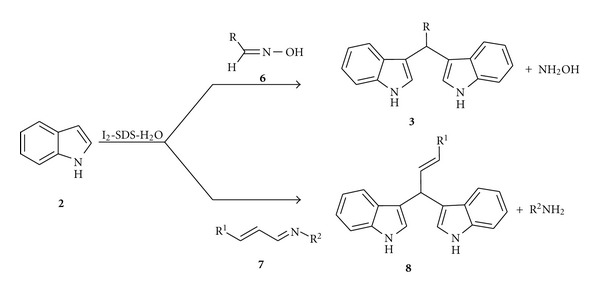
Reaction of azabutadienes and aldoximes with indoles.

To study the scope and limitations of the reaction, I_2_-SDS-H_2_O system was applied to the reaction of indole (**2**) and 2,3-diaza-1,3-butadiene derivatives (Table 2, entries a–f). The bisindoles were formed in excellent yields under the reaction condition. It was observed that the reaction was relatively faster when an electron withdrawing substituent, for example, NO_2_, was present in the phenyl ring of the 2,3-diaza-1,3-butadienes (Table 2, entry e) in comparison to the electron donating groups, for example, OMe and OH (Table 2, entries b and f). Identical results were obtained when 2-methylindole (**2a**) was used in place of indole (**2**) (Table 2, entries g and h). All the products were characterized by their IR, ^1^H NMR, ^13^C NMR, and mass spectral data and also by comparison with the literature report (Scheme 2) [24, 25].

In order to further explore the efficiency of the I_2_-SDS-H_2_O system the reaction of the oximes **6** and indoles **2** was studied. When oximes (1 mmol) and indole (2 mmol) were allowed to react under the same reaction condition described earlier, *bis*-indolylalkanes formed (Table 3). The product was filtered off and the filtrate was treated with benzaldehyde in ethanol. The resulted product was Benzaldoxime, which proved the liberation of hydroxylamine during the reaction. It was found that no Michael addition product [37] was formed and only a trace amount of indole dimer **5** [35] could be identified. The system was also applied to the reaction of 1-aza-1,3-butadienes **7** with indole (**2**) which produced bis (indolyl)alkanes **8** in very good yield eliminating aryl amine in the reaction mixture (Table 4). All the products were well characterized by comparison of their spectral and mass data with that of the reported value [24, 25].

## 3. Conclusions

In conclusion, we have shown the dual catalytic activity of I_2_-SDS-H_2_O system which deprotects the azadienes, oximes, and azabutadienes and produces bis (alkyl)indoles *in situ* when indole is present in the reaction medium. The two-step reaction can be carried out without using acid, transition metals, and organic solvents. Besides, the reaction condition is mild and can be done in water under neutral condition which contributes to the criteria of green chemistry.

## 4. Experimental

Melting points were measured using Buchi B-540 apparatus and are uncorrected. ^1^H NMR spectra were recorded on Avance DPX 300 MHz FT-NMR spectrometer. Chemical shifts are expressed in *δ* units relative to tetramethylsilane (TMS) signal as internal reference. IR spectra were recorded on FT-IR-system-2000 Perkin Elmer spectrometer on KBr pellets or in CHCl_3_. Mass spectra were recorded on ESQUIRE 3000 Mass Spectrometer. All reagents were obtained from commercial sources and used without further purification. The solvents for chromatography were distilled before use.

### 4.1. General Procedure for the Synthesis of 3,3′-Bis(indolyl)alkanes

In a 50 mL round bottom flask, 15 mol% of I_2_ was first dissolved in water (10 mL). 2,3-Diaza-1,3-butadiene (1 mmol) and indole (4 mmol) were added and stirred in the presence of sodium dodecyl sulphate (SDS) (0.02 g) for the stipulated time. The progress of the reaction was monitored by TLC. The product formed was filtered off and washed with water, dried, and recrystallized from ethanol. 

Identical reaction condition was followed when 1-aza-1,3-butadienes and oximes were used as reactants. In this case, 2 mmol of indoles were used to react with 1 mmol of imines.

### 4.2. 3,3′-Bis(indolyl)phenylmethane **(3a)** [24]

Colorless solid; mp: 150–152°C; FTIR (KBr): *ν* 3418, 3058, 1623, 1611, 1445, 1093 cm^−1^; ^1^H NMR (300 MHz, CDCl_3_): *δ* 5.95 (s, 1H, Ar–CH), 6.73(s, 2H), 7.06 (t, 2H, *J *= 6.8 Hz), 7.18–7.27 (m, 3H), 7.31–7.36 (m, 2H), 7.36–7.42 (m, 6H), 7.98 (br, s, 2H, NH); ^13^C NMR (75 MHz, CDCl_3_): 40.7, 111.2, 119.1, 119.5, 120.4, 122.1, 123.8, 126.9, 126.9, 128.2, 129.1, 136.8, 144.8; HRMS calcd for C_23_H_18_N_2_ (M^+^): 322.2851, found 322.2832; Anal.calcd.: C, 85.70; H, 5.59; N, 8.69; found C, 85.75; H, 5.56; N, 8.56.

### 4.3. 3,3′-Bis(indolyl)-4-chlorophenylmethane **(3d)** [24]

 Pink solid; mp: 76-77°C; FTIR (KBr): *ν* 3415, 3060, 1491, 1465, 1095 cm^−1^; ^1^H NMR (300 MHz, CDCl_3_): *δ* 5.91 (s, 1H, Ar–CH), 6.76 (s, 2H), 7.08 (t, 2H, *J* = 8.3 Hz), 7.22 (t, 2H, *J* = 7.9 Hz), 7.28–7.42 (m, 8H), 8.01 (br, s, 2H, NH); ^13^C NMR (75 MHz, CDCl_3_): 39.8, 111.5, 122.6, 123.8, 127.1,128.4, 129.8, 130.1, 130.9, 131.0, 131.6, 137.2, 143.5; HRMS calcd for C_23_H_17_N_2_Cl (M^+^): 356.7371; found 356.7324; Anal.calcd.: C, 77.52; H, 4.77; N, 7.86; found C, 77.48; H, 4.72; N, 7.80.

### 4.4. 3,3^*΄*^-Bis(indolyl)furylmethane **(3j)** [24]

 Brown solid; mp: 323–325°C; FTIR (KBr): *ν* 3420, 1720, 1455, 1258 cm^−1^; ^1^H NMR (300 MHz, CDCl_3_): *δ* 5.98 (s, 1H, Ar-CH), 6.85 (s, 2H), 7.10–7.55 (m, 11H), 8.05 (br, s, 2H, NH), ^13^C NMR (75 MHz, CDCl_3_): 34.8, 107.0, 110.0, 111.5, 117.8, 119.9, 120.0, 122.5, 124.8, 127.1, 136.8, 142.2; HRMS calcd for C_21_H_16_N_2_O_2_ (M^+^): 312.2621; found 312.2611; Anal.calcd. C, 80.84; H, 5.12; N, 8.97; found C, 84.05; H, 5.15; N, 8.94.

All the products were fully characterized by ^1^H and ^13^C NMR and MS analyses. The spectral data of the compounds are available in the Supplementary Material available online at doi: 105402/2012/635835.

## Supplementary Material

The supplementary material contains all the characterization data (Melting point, IR, 1H NMR, 13C NMR, HRMS, and CHN analysis) of the compounds 3a-3g, 3j, and 8.

## Figures and Tables

**Table 1:** Optimization of the reaction conditions^a^.

Entry	Reagent system	Time (h)	Yields^b^ (%)
1	I_2_-SDS-H_2_O	3.5	93
2	I_2_-Triton X-H_2_O	7.0	74
3	I_2_-Aliquat 336-H_2_O	8.0	72
4	I_2_-CTAB-H_2_O	6.3	69
5	No catalyst	24.0	—

^a^The reactions were carried out using 1 mmol of 1,4-diphenyl-2,3-diaza-1,3-butadiene (**1a**) with 4 mmol of indole (**2a**) in presence of 15 mol% of I_2_ and 0.02 g of surfactant.

^b^Isolated yield.

**Table 2:** Reaction of indoles with 2,3-diaza-1,3-butadienes.

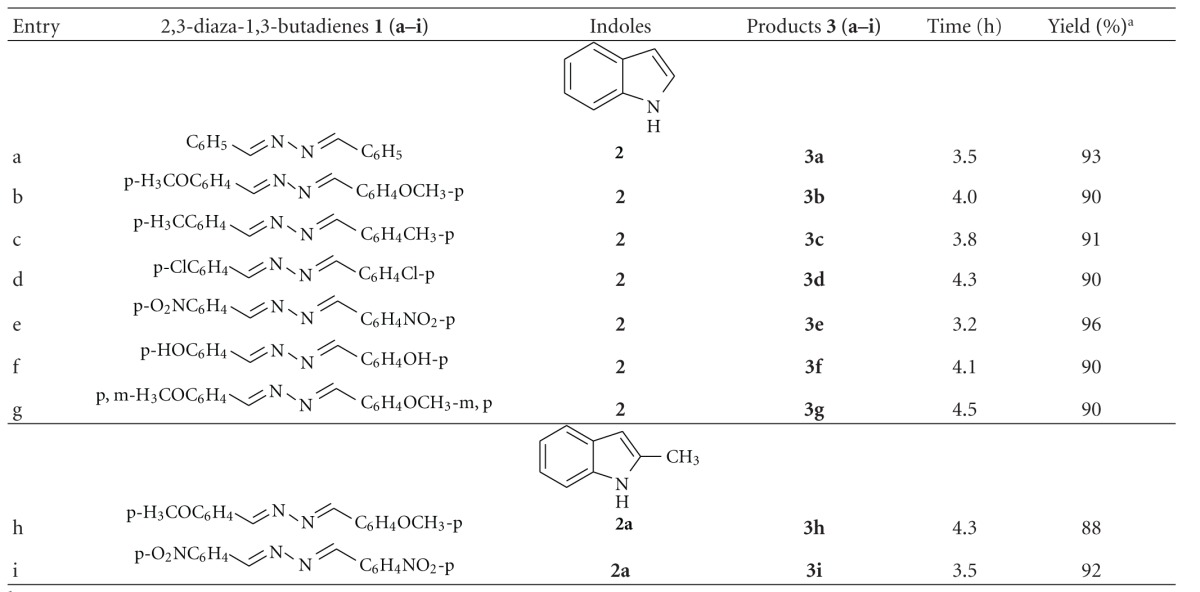

^a^Isolated yield.

**Table 3:** Reaction of indoles with aldoximes.

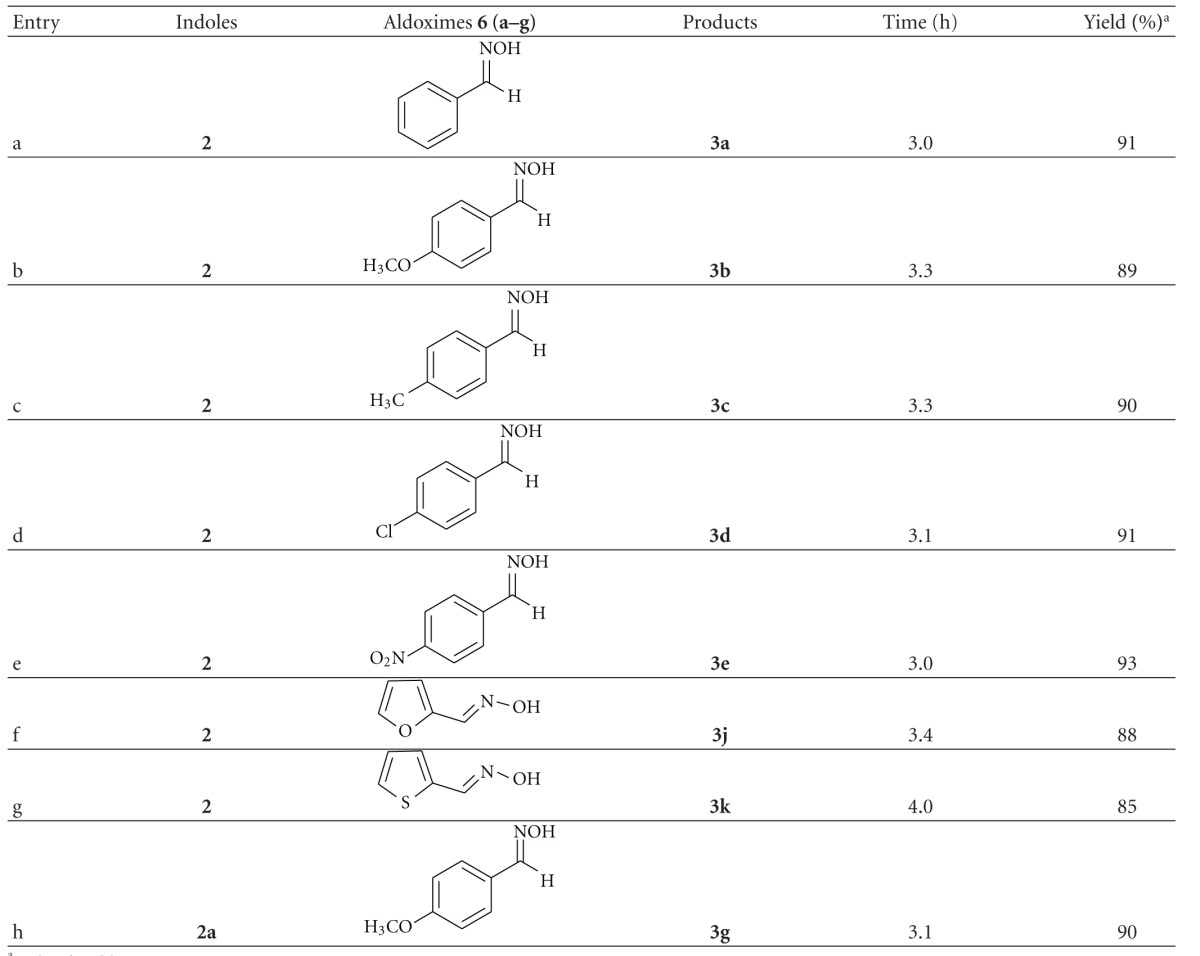

^a^Isolated yield.

**Table 4:** Reaction of 1-azabutadienes with indole (**2**).

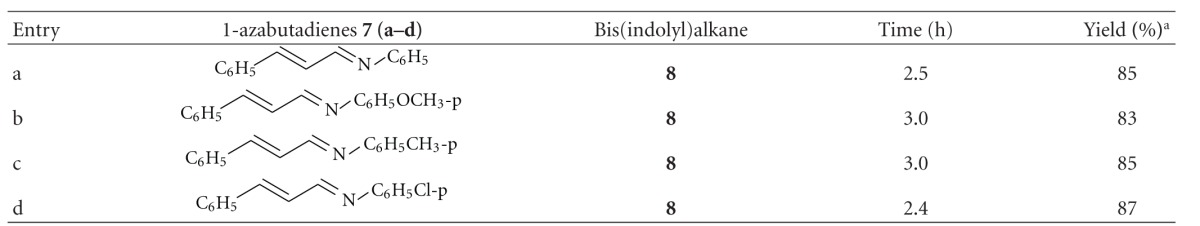

^a^Isolated yield.
