# Supplementary material for: I2-SDS-H2O System: A highly Efficient Dual Catalytic Green System for Deprotection of Imines and in Situ Preparation of Bis(indolyl)alkanes from Indoles in Water
Source: ISRN Org Chem. 2012 Aug 27;2012:635835. doi: 10.5402/2012/635835 (PMC3767350; doi:10.5402/2012/635835)
Supplement: Supplementary file 1 — The supplementary material contains all the characterization data (Melting point, IR, 1H NMR, 13C NMR, HRMS, and CHN analysis) of the compounds 3a-3g, 3j, and 8. [file 635835.f1.pdf]

Supporting Informations

I_2-SDS-H_2O System: A highly efficient dual catalytic green system for deprotection of imines and *in situ* preparation of *bis*-indolylalkanes from indoles in water

Parasa Hazarika,[a] Pallab Pahari,[b] Manash Jyoti Borah,[b] and Dilip Konwar[b]*

[a]*Departmnet of Chemistry, Jorhat Institute of Science and Technology,*

Jorhat-785010, Assam, India

[b]*Synthetic Organic Chemistry Division, North East Institute of Science and Technology (a constituent establishment of CSIR, India), Jorhat-785006, Assam, India*.

E-mail: dkonwar@yahoo.co.uk

Fax: +913762370011; Tel: +913762370012

Table of contents

Compound characterization data --- S2

References --- S7

Compound characterization data

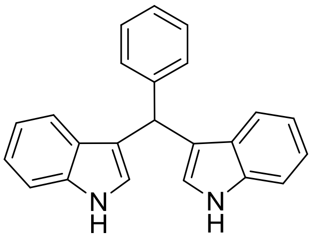

3a

3,3´-Bis(indolyl)phenylmethane (3a) [1]

Dark red solid; mp:150-152 °C; FTIR (KBr): ν 3418, 3058, 1623, 1611, 1445, 1093 cm^{-1}; ^{1}H NMR (300 MHz, $CDCl_3$): δ 5.95 (s, 1H, Ar-CH), 6.73(s, 2H), 7.06 (t, 2H, *J* = 6.8 Hz), 7.18-7.27 (m, 3H), 7.31-7.36 (m, 2H), 7.36-7.42 (m, 6H), 7.98 (br, s, 2H, NH); ^{13}C NMR (75MHz, $CDCl_3$): 40.7, 111.2, 119.1, 119.5, 120.4, 122.1, 123.8, 126.9, 126.9, 128.2, 129.1, 136.8, 144.8; HRMS calcd for $C_{23}H_{18}N_2$ (M^+): 322.2851, found 322.2832; Anal.calcd.: C, 85.70; H, 5.59; N, 8.69; found C, 85.75; H, 5.56; N, 8.56.

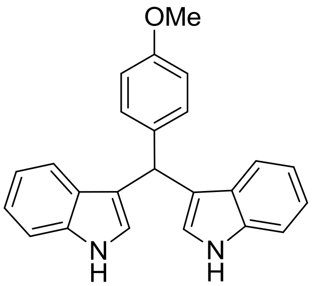

3b

3,3´-Bis(indolyl)-4-methoxyphenylmethane (3b) [1]

Brown solid; mp: 187 °C; FTIR (KBr): ν 3423, 2919, 1625, 1516, 1458, 1254, 1228 cm^{-1}; ^{1}H NMR (300 MHz, $CDCl_3$): δ 3.84 (s, 3H, CH_3), 5.91 (s,1H, Ar-CH), 6.70 (s, 2H), 6.89 (d, 2H, *J* = 8.3 Hz), 7.09 (t, 2H, *J* = 7.3 Hz), 7.21 (t, 2H, *J* = 7.3 Hz), 7.29 (s, 2H), 7.38-7.46 (m, 4H), 8.01 (br, s, 2H, NH); ^{13}C NMR (75MHz, $CDCl_3$): 39.4, 56.5, 111.2,

113.9, 118.2, 119.9, 122.1, 129.2, 126.8, 136.8; HRMS calcd for $C_{24}H_{20}N_2O$ (M^+): 352.2981, found 352.2942; Anal.calcd.: C, 81.81; H, 5.68; N, 7.95; found C, 81.72; H, 5.78; N, 7.98.

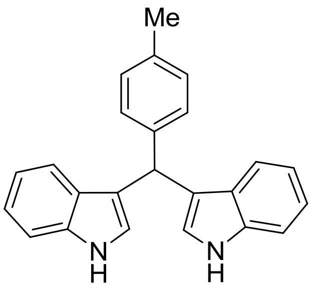

3c

3,3´-Bis(indolyl)-4-methylphenylmethane (3c) [1]

solid, mp: 94-95 °C; FTIR (KBr): ν 760, 105, 1205, 118, 1604, 2956, 3044, 3117, 3450 cm^{-1}; ^{1}H NMR (300 MHz, $CDCl_3$): δ 2.35 (s, 3H), 5.82 (s, 1H), 6.72 (d, 2H, *J* = 2.4 Hz), 7.01 (t, 2H, *J* = 8.0 Hz), 7.3 (d, 2H, *J* = 8.3 Hz), 7.81 (br, s, 2H, NH); HRMS calcd for $C_{24}H_{20}N_2$ (M^+): 336.6785; found 336.6754; Anal.calcd. C, 85.54; H, 5.94; N, 8.31; found C, 85.50; H, 5.98; N, 8.33.

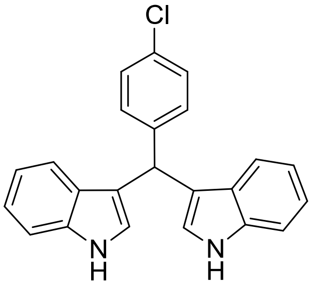

3d

3,3´-Bis(indolyl)-4-chlorophenylmethane (3d) [1]

Pink solid; mp: 76-77 °C; FTIR (KBr): ν 3415, 3060, 1491, 1465, 1095 cm^{-1}; ^{1}H NMR (300 MHz, $CDCl_3$): δ 5.91 (s, 1H, Ar-CH), 6.76 (s, 2H), 7.08 (t, 2H, *J* = 8.3 Hz), 7.22 (t, 2H, *J* = 7.9 Hz), 7.28-7.42 (m, 8H), 8.01 (br, s, 2H, NH); ^{13}C NMR (75MHz, $CDCl_3$):

39.8, 111.5, 122.6, 123.8, 127.1,128.4, 129.8, 130.1, 130.9, 131.0, 131.6, 137.2, 143.5; HRMS calcd for $C_{23}H_{17}N_2Cl$ (M^+): 356.7371; found 356.7324; Anal.calcd.: C, 77.52; H, 4.77; N, 7.86; found C, 77.48; H, 4.72; N, 7.80.

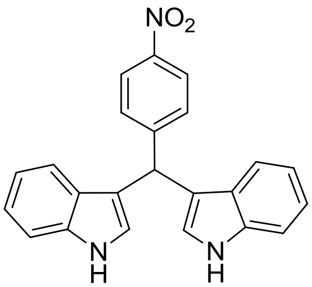

3e

3,3´-Bis(indolyl)-4-nitrophenylmethane (3e) [1]

Brown solid; mp: 217-219 °C; FTIR (KBr): ν 3415, 2917, 1595, 1515, 1456, 1344 cm^{-1}; ^1H NMR (300 MHz, $CDCl_3$): δ 6.05 (s, 1H, Ar- CH), 6.81 (s, 2H), 7.05-7.11 (m, 3H), 7.39 (d, 3H, *J* = 8.0 Hz), 7.47 (d, 2H, *J* = 8.0Hz), 7.60 (d, 2H, *J* = 8.8 Hz), 8.10 (br, s, 2H, NH), 8.21 (d, 2H, *J* = 8.8 Hz); HRMS calcd for $C_{23}H_{17}N_3O_2$ (M^+): 367.2911; found 367.2921. Anal.calcd.: C, 75.20; H, 4.63; N, 11.44; found C, 75.25; H, 4.65; N, 11.60.

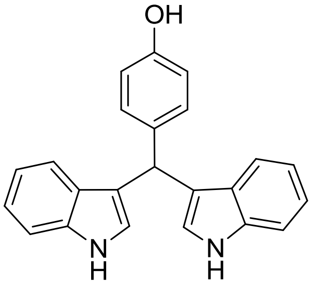

3f

3,3´-Bis(indolyl)-4-hydroxyphenylmethane (3f) [1]

Pink solid; mp: 208 °C; ^1H NMR (300 MHz, DMSO): δ 9.61 (s, 2H, NH), 8.59 (s, H, OH), 7.24 (t, 4H, J = 7.7 Hz), 7.10 (d, 2H, *J* = 7.4 Hz), 7.05 (t, 2H, *J* = 7.4 Hz), 6.80 (t, 2H, *J* = 7.3 Hz), 6.62 (d, 4H, *J* = 8.73 Hz), 5.67 (s, 1H); ^{13}C NMR

(75MHz, $CDCl_3$): 160.2, 142.2, 140.4, 134.5, 132, 129.2, 126.5, 124.5, 124.3, 123.3, 120.1, 116.1, 44.5; HRMS calcd for $C_{23}H_{18}N_2O$ (M^+): 338.5676; found 338.5642; Anal.calcd.: C, 81.56; H, 5.31; N, 8.27; O, 4.72; found C, 81.60; H, 5.25; N, 8.22; O, 4.77.

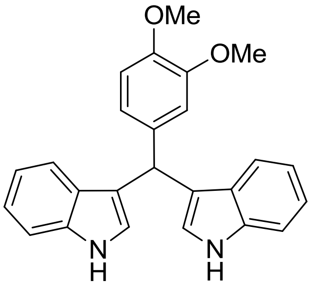

3g

3,3´-Bis(indolyl)-3,4- dimethoxyphenylmethane (3g)

solid, mp: 197-199 ^{o}C; FTIR (KBr): ν 3455, 3065, 2982, 1622, 1485, 1235, 1006, 763 cm^{-1}; ^1H NMR (300 MHz, $CDCl_3$): δ 3.65 (s, 3H), 3.83 (s, 3H), 5.70 (s, 1H), 6.65 (d, 2H, *J* = 2.2 Hz), 6.75 (d, 2H, *J* = 8.05 Hz), 6.80 (s, 1H), 6.86 (d, 2H, *J* = 8.05 Hz), 7.10 (t, 2H, *J* = 8.05 Hz), 7.26 (t, 4H, *J* = 8.03 Hz), 10.40 (brs, 2H, NH); HRMS calcd for $C_{25}H_{22}O_2N_2$ (M^+): 382.5645, found 382.5632; Anal. calcd. C, 78.41; H, 5.75; N, 7.31; O, 8.36; found C, 78.45; H, 5.71; N, 7.30; O, 8.37.

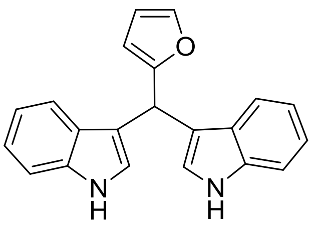

3i

3,3´-Bis(indolyl)furylmethane (3j) [1]

Brown solid; mp: 323-325 °C; FTIR (KBr): ν 3420, 1720, 1455, 1258 cm^{-1}; ^{1}H NMR (300 MHz, $CDCl_3$): δ 5.98 (s, 1H, Ar-CH), 6.85 (s, 2H), 7.10-7.55 (m, 11H), 8.05 (br, s, 2H, NH), ^{13}C NMR (75MHz, $CDCl_3$): 34.8, 107.0, 110.0, 111.5, 117.8, 119.9, 120.0, 122.5, 124.8, 127.1, 136.8, 142.2; HRMS calcd for $C_{21}H_{16}N_2O_2$ (M^+): 312.2621; found 312.2611; Anal.calcd. C, 80.84; H, 5.12; N, 8.97; found C, 84.05; H, 5.15; N, 8.94.

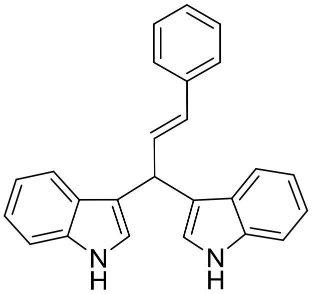

8

3,3´-Bis(indolyl)-3-phenyl-2-propene (8) [2]

Gummy; FTIR (KBr): ν 3461, 3120, 2962, 1571, 1463, 1055 cm^{-1}; ^{1}H NMR (300 MHz, $CDCl_3$): δ 5.95 (s, 1H), 6.51 (d, 1H, J = 16.6 Hz), 6.68 (d, 2H, J = 2.3 Hz), 7.00 (t, 2H, J = 8.1 Hz), 7.18 (t, 2H, J = 8.1 Hz), 7.30 (m, 4H), 7.38 (m, 3H), 7.61 (m, 2H), 7.81 (d, 1H,

J = 16.6 Hz), 7.92 (br, s, 2H, NH); HRMS calcd for $C_{25}H_{20}N_2$ (M^+): 348.3090; found 348.3051; Anal.calcd.: C, 78.12; H, 5.74; N, 8.01; found C, 78.20, H, 5.68; N, 8.03.

References

[1] S. J. Ji, S. Y. Wang, Y. Zhang, T. P. Loh, "Facile synthesis of *bis*-(indolyl)methanes using catalytic amount of iodine at room temperature under solvent-free conditions," *Tetrahedron* vol. 60, no. 9, pp. 2051-2055, 2004.

[2] B. P. Bandgar and K. A. Shaikh, "Molecular iodine-catalyzed efficient and highly rapid synthesis of bis(indolyl)methanes under mild conditions," *Tetrahedron Letters*, vol. 44, no. 9, pp. 1959-1961, 2003.
